# Single-Cell Multiomics Techniques: From Conception to Applications

**DOI:** 10.3389/fcell.2022.854317

**Published:** 2022-03-21

**Authors:** Maria A. Dimitriu, Irina Lazar-Contes, Martin Roszkowski, Isabelle M. Mansuy

**Affiliations:** Laboratory of Neuroepigenetics, Brain Research Institute, University of Zurich and Institute for Neuroscience, ETH Zurich, Zurich, Switzerland

**Keywords:** single-cell, multiomics, genomics, epigenomics, transcriptomics, chromatin accessibility

## Abstract

Recent advances in methods for single-cell analyses and barcoding strategies have led to considerable progress in research. The development of multiplexed assays offers the possibility to conduct parallel analyses of multiple factors and processes for comprehensive characterization of cellular and molecular states in health and disease. These technologies have expanded extremely rapidly in the past years and constantly evolve and provide better specificity, precision and resolution. This review summarizes recent progress in single-cell multiomics approaches, and focuses, in particular, on the most innovative techniques that integrate genome, epigenome and transcriptome profiling. It describes the methodologies, discusses their advantages and limitations, and explains how they have been applied to studies on cell heterogeneity and differentiation, and epigenetic reprogramming.

## 1 Introduction

Complex tissues and organisms are formed by a heterogeneity of cells that divide, proliferate and differentiate, and go through various physiological states during development and in adulthood (Mayr et al., 2019). Although the fate of each cell is intrinsically determined, it is strongly influenced by cell-cell interactions and by external factors. The processes taking place within each individual cell are a result of complex interactions between chromatin, transcripts and proteins. Whether a coding or non-coding transcript is expressed in a cell at each given time, but also which isoforms and splice variants become synthesized, is regulated by a combination of genetic and epigenetic factors, including transcription factors, chromatin remodelers, but also non-coding RNAs and transposable elements (Philpott et al., 2020). To identify these activity-dependent regulatory processes, most approaches in the past used transcriptomic and/or proteomic methods of analyses on whole tissues or cell suspensions. Although useful, such bulk approaches are not ideal because they average information derived from thousands or millions of cells, which masks cell-specific features or features typical of developmental processes such as lineage choice (Hu et al., 2018).

The development of methods for single-cell analyses greatly helps to address this limitation. Single-cell sequencing has recently been adopted by multiomic strategies, which bring together simultaneous information about different molecular modalities and their relationship in individual cells. Multiomics combines the assessment of cellular features pertaining to the genome (e.g., chromatin accessibility, copy-number variations), the transcriptome, the proteome and, more recently, spatial patterns of gene expression. Such a multiomic approach overcomes the drawback of correlative integration of unimodal datasets obtained from separate experiments (Ma Anjun et al., 2020) and identifies key molecular features within individual cells. In the past decade, single-cell sequencing technologies have been increasingly used to study the cellular heterogeneity of tissues and organisms during development and in adulthood. Thus, multiomics strategies and single-cell sequencing platforms are powerful methods that, together, can uncover functional cellular diversity at a large scale, through a more mechanistic dissection of dynamic cell states (Strzelecka et al., 2018; Lee et al., 2020).

While the first single-cell multiomics (sc-multiomics) techniques were only published in 2014 (Han et al., 2014), the methods have since then rapidly diversified, with new approaches showing improvement in throughput, sequencing depth, coverage, but also with potential for automation. In this review, we cover recent advances in sc-multiomics technologies and focus on methods that simultaneously profile the genome, epigenome, and/or transcriptome in single cells. We further discuss their current and potential applications towards understanding gene regulation in development and disease.

### 2 The Fundamentals of Single-Cell Multiomics

All sc-multiomics techniques involve processing tissues to a single cell suspension, isolating the individual cells, lysing the cells in a manner that preserves the stability and integrity of subcellular components (DNA, RNA, proteins) and, finally, processing these molecules to obtain libraries for sequencing.

### 2.1 Single-Cell Isolation

**FIGURE 1:**
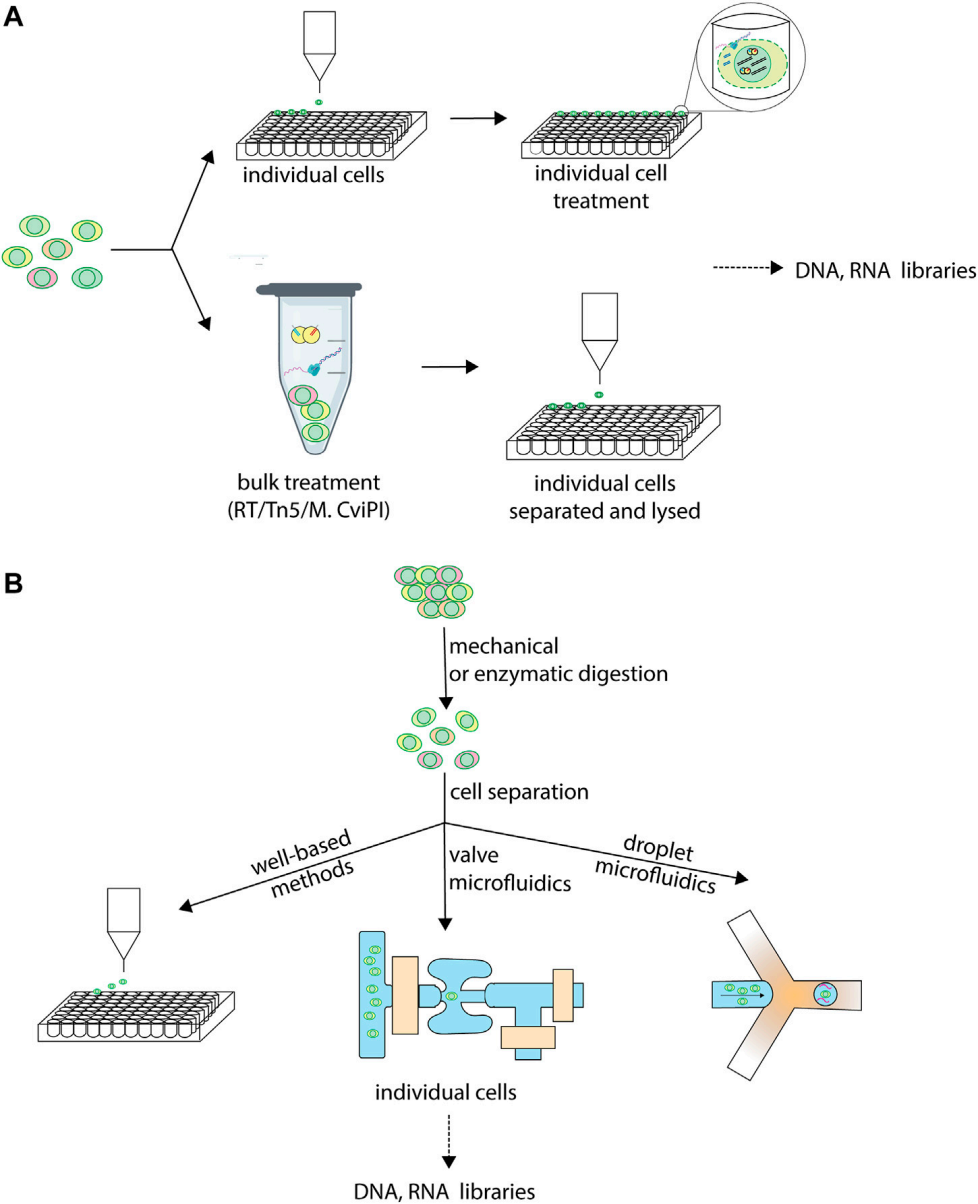
Individual cell isolation in sc-multiomics. Single-cell multiomics techniques differ in when and how, during their workflow, they isolate single cells into individual reactions for further processing. **(A)** Different stages at which cells are isolated in sc-multiomics. While some sc-multiomics techniques 1) start from the single-cell suspension by first isolating individual cells (using one of the approaches in **(B)** and only then subject cells individually to further processing, such as enzymatic treatments (top), other techniques 2) subject the single-cell suspension to enzymatic treatment first and only then isolate single cells (bottom). **(B)** Different technologies for isolating individual cells in sc-multiomics. Well-based methods rely on techniques such as fluorescence-activated cell sorting (FACS) to sort individual cells into unique wells. Valve microfluidics methods treat single cells individually in unique reaction wells on microfluidic chips. Droplet microfluidics encapsulate individual cells into unique barcoded droplets for further processing.

Similarly to mono-omic single-cell techniques, all sc-multiomics techniques must achieve the physical isolation of single cells into discrete reaction compartments. This is necessary to enable the retracing during sequencing data analysis of which cell gave rise to which sequencing reads. Some techniques isolate single cells at the start of their workflow and then subject individual cells to further processing (Figure 1A, top). Other techniques partially process the whole single-cell suspension in a first reaction by subjecting it to DNA tagmentation or RNA reverse transcription, often including a first barcoding event, and only subsequently isolate individual cells into separate reactions before further processing (Figure 1A, bottom).

The sc-multiomic techniques reviewed here can be catalogued into three main categories on the basis of how they approach single cell separation: well-based techniques, continuous-flow microfluidics-based techniques and droplet-microfluidics-based techniques (Figure 1B). Well-based techniques use multi-well plates, in which each cell is distributed to one well containing a unique molecular identifier that becomes attached to the molecules of interest in each well. The distribution of cells to individual wells is most frequently achieved by fluorescence-activated cell sorting (FACS), by limiting dilution or by manual cell picking. Other methods rely on microfluidics, a fluid-handling technology using microscale devices that allows fast and precise manipulation of small volumes of sample, reducing reagent costs (Sackmann et al., 2014). Because they are miniaturized, microfluidics methods considerably reduce reaction volumes, requiring nanoliters, or even picoliters, of reagents compared to microliters for microwell plates. On the one hand, this minimizes reagent- and environment-borne contaminants (Nishikawa et al., 2015). On the other hand, it leads to a molecular crowding effect, whereby the template is more concentrated in the reaction environment, thus minimizing amplification bias, which ultimately increases the accuracy and precision of the measurements (Ballantyne et al., 2006).

Two distinct microfluidics methodologies have been used for single-cell omics analyses. First, continuous-flow microfluidics uses chip valves to trap single cells in discrete reaction chambers of nanoliter volume where each step of the workflow occurs, allowing for automation of the entire process (Shim et al., 2013; Philpott et al., 2020). Second, droplet-based microfluidics co-encapsulates single cells with barcoded beads into microdroplets (Macosko et al., 2015). Valve-based systems afford more versatility for adding reagents, thus allowing more steps to be conducted on-chip, including the reverse transcription and amplification reactions, leading to a less labor-intensive workflow and increased precision. However, droplet systems afford higher cell capture efficiencies and an order of magnitude higher throughputs, since droplets can be generated on-demand and on a large scale, unlike chambers (Gao et al., 2019).

### 2.2 Processing of Intracellular Components

**FIGURE 2:**
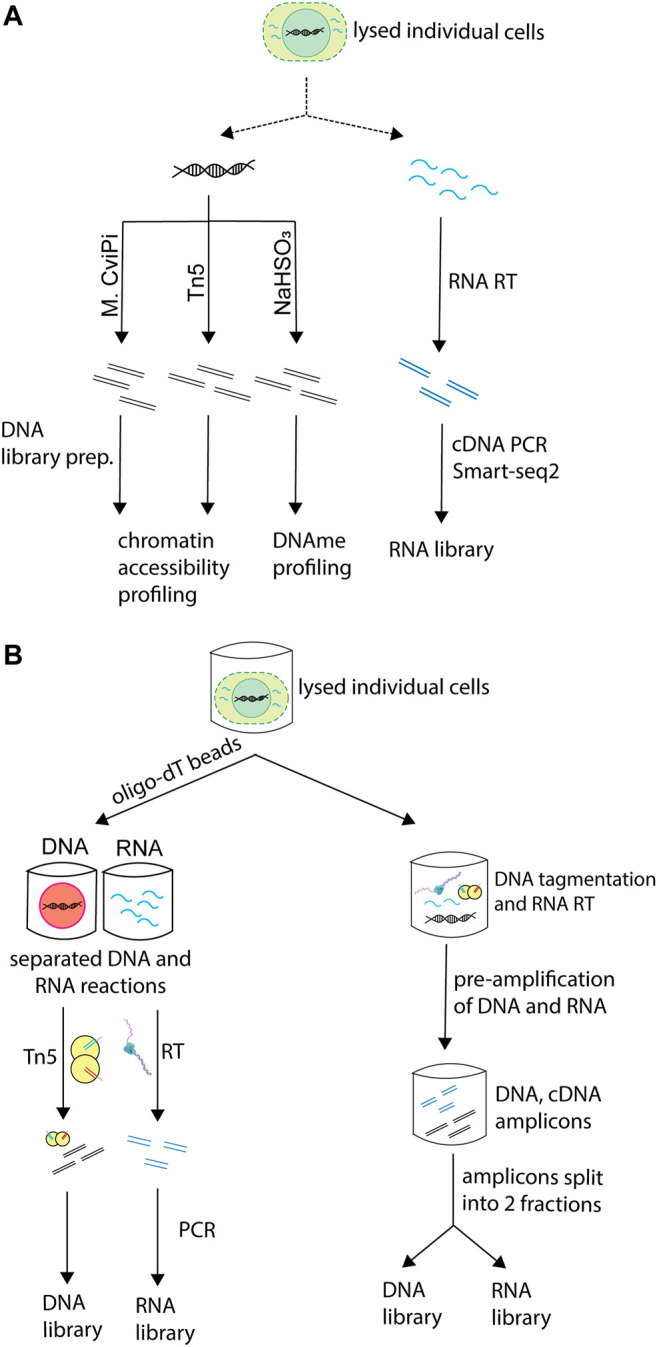
Processing of different molecular layers in sc-multiomics. Single-cell multiomics techniques that simultaneously profile the genome and transcriptome of cells differ in the way they process these molecules. **(A)** Different types of processing of DNA and RNA employed in sc-multiomics depending on the goal of the technique. sc-multiomics techniques differ in the way they process DNA and/or RNA, depending on what the approach aims to profile: DNA can be either treated with M. CviPI (a DNA methyltransferase that methylates cytosine in accessible GC dinucleotides) or with Tn5 (if the aim is to characterize chromatin status), or bisulfite converted (if the aim is DNA methylation profiling), while RNA is subjected to RT and amplification. **(B)** Simultaneous or parallel processing of DNA and RNA in sc-multiomics. In sc-multiomics, once individual cells are lysed, either 1) DNA and RNA can be first separated from each other using oligo-dT beads and then processed in parallel (left), or 2) the lysed cell can be subjected to DNA tagmentation and RNA reverse transcription (RT) in one reaction, followed by simultaneous pre-amplification of the DNA and cDNA, and then splitting of amplicons into fractions for library preparations (right).

Depending on the molecular information each technique aims to provide, techniques vary in which molecules they process and which treatments they subject the subcellular components to (Figure 2A). Some of the techniques that profile chromatin accessibility extract the gDNA and treat it with a DNA methyltransferase, which methylates cytosines in accessible GC dinucleotides, thus marking accessible chromatin. Others subject the gDNA to tagmentation by the Tn5 transposase, which fragments chromatin at accessible sites, thus providing the same type of information. Approaches that aim to profile DNA methylation rely on bisulfite conversion of the gDNA.

Among the multiomics techniques that simultaneously profile the genome and transcriptome of single cells, some methods separate DNA and RNA immediately after cell lysis and before any processing (Figure 2B, left). This can be done by taking advantage of their physical properties (e.g., different densities through centrifugation) or sequence characteristics, for instance the presence of polyadenylated (polyA+) sequences in messenger RNA and some non-coding RNAs. Other methods subject the whole lysate containing DNA and RNA to reverse-transcription and pre-amplification. Then, the reaction is split into two aliquots, one used for gDNA processing and the other for RNA library preparation (Figure 2B, right) (Dey et al., 2015; Lee et al., 2020).

sc-multiomics techniques also vary in the populations of RNA they profile. While most capture all polyA + RNA, some capture only nuclear polyA + RNA (single-nucleus chromatin accessibility and mRNA expression profiling, SNARE-seq (Chen et al., 2019)) or polyA- RNA (simultaneous isolation and sequencing of genomic DNA and total RNA, scSIDR-seq (Han et al., 2018)). Further, depending on the strategy used to prepare RNA libraries, techniques vary in their transcript sequence coverage, and thus in their applicability. For instance, techniques based on the Switching Mechanism At the 5′-end of RNA Transcripts (Smart-seq) (Ramsköld et al., 2012) RNA library preparation strategy, including single-cell targeted mutational analysis and parallel RNA sequencing (TARGET-seq (Rodriguez-Meira et al., 2019)), simultaneous chromatin accessibility and gene expression profiling (sc (ATAC + RNA)-seq (Reyes et al., 2019a)), single-cell chromatin accessibility and transcriptome sequencing (scCAT-seq (Liu et al., 2019)), single-cell nucleosome, methylation and transcription sequencing (scNMT-seq (Clark et al., 2018)) and methods relying on the Multiple Annealing and dC-Tailing-based Quantitative (MATQ-seq) (Sheng et al., 2017) library preparation strategy, such as single-cell nucleosome occupancy, methylome and RNA expression sequencing (scNOMeRe-seq (Wang et al., 2021)), recover the whole transcript, allowing for applications such as splice-variant analysis. In contrast, protocols such as Droplet Based scRNA-Seq (Drop-seq) (Macosko et al., 2015) are restricted to the 3′-end of the transcript (SNARE-seq) (Chen et al., 2019) and do not capture the 5′-end of the transcript, precluding the ability to gain information about transcription start sites, splice variants and 5′ untranslated regions (Haque et al., 2017). However, in cases where throughput needs to be high and sensitivity is not crucial, costs can be decreased by using 3′ end approaches such as Drop-seq (Macosko et al., 2015).

### 2.3 Barcoding

**FIGURE 3:**
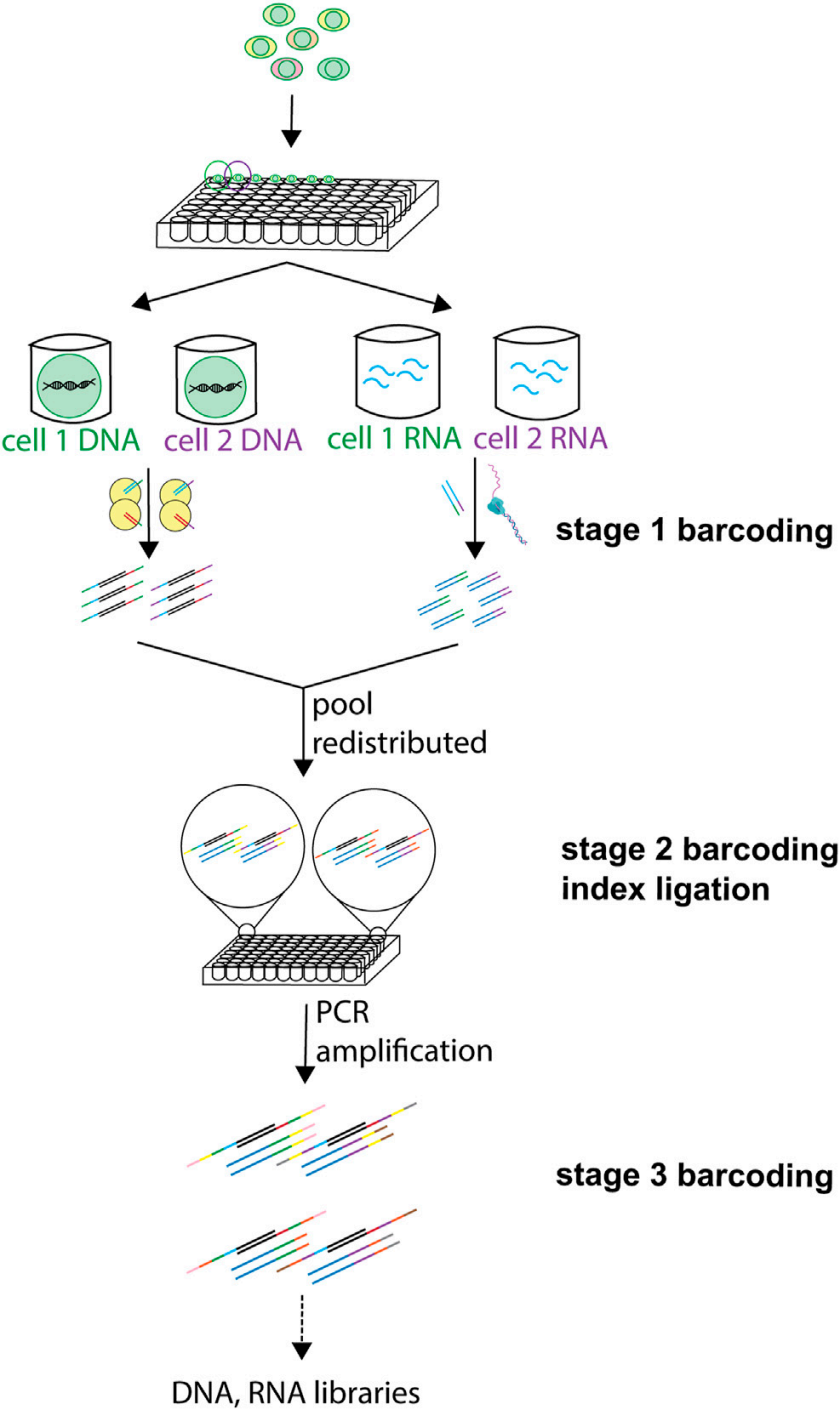
Use of barcoding at different steps in sc-multiomics. Barcoding of DNA and RNA can be employed at different steps of the processing of single cells, and different labels and techniques can be used at one or more of these steps. A first barcode can be inserted in DNA during tagmentation and in RNA during RT. Then, after pooling of samples and redistribution into wells, one or more ligation-based barcoding is possible. A third barcoding is possible during amplification.

Barcoding (or indexing) involves attaching a unique oligonucleotide sequence to the molecules of interest from 1 cell, thereby generating a unique cellular identifier. Cell-specific differential barcoding allows identifying each cell individually and thus offers the possibility to pool cells with different barcodes (Philpott et al., 2020) into one sample. This permits to subsequently demultiplex the reads resulting from a pooled library to its cells of origin following sequencing. Barcodes can be added during tagmentation of the gDNA, reverse transcription of the RNAs, amplification of both, or by ligation. sc-multiomics methods reviewed here apply barcoding at different steps in their workflow and some even apply combinatorial indexing approaches, in which different rounds of barcoding are employed in the same experiment (Figure 3). Because of the need to uniquely compartmentalize individual cells, most well-based methods have low throughput. However, the use of combinatorial indexing strategies can increase the throughput of well-based methods (sciCAR (Cao et al., 2018), Paired-seq (Zhu et al., 2019)) by several orders of magnitude (Vitak et al., 2017). Because populations of cells are indexed together at each step, then pooled and redistributed in the subsequent round, each cell passes through a unique combination of wells (and barcodes), allowing the compartmentalization of each cell to occur virtually, through the unique combination of barcodes they receive at the end of the barcoding rounds, rather than physically (Cusanovich et al., 2015).

An ideal sc-multiomics method should have a simple workflow and be highly sensitive and accurate while having low background. It should minimize potential biases and avoid confounding parameters, like for instance amplification bias. At the same time, it should provide high efficiency such as high library complexity for each measured factor e.g., DNA or RNA sequence. sc-multiomics methods should also ideally be high throughput, to allow for complex projects such as devising cell atlases. The workflow of an efficient sc-multiomics technique should involve as many reactions in bulk as possible to minimize reagent costs, while maximizing throughput (Kebschull and Zador, 2018).

## 3 Multiomics Characterization of Chromatin Structure, Transcriptome and Epigenome at Single-Cell Resolution

sc-multiomics approaches provide researchers with a powerful and direct way to characterize the coordination between layers of genomic regulation in individual cells. The ability to profile relationships between different aspects of the epigenome in single cells is particularly useful for the study of highly dynamic epigenetic patterns, as occur during gamete and embryonic development, and during cell differentiation events. Leveraging the power of such approaches to study gamete maturation, Gu *et al.* found that chromatin opening at promoters precedes *de novo* gene body methylation before oocyte growth initiation (Gu et al., 2019a). Applying similar approaches to study mouse embryonic development, Guo *et al.* and Wang *et al.* found allelic differences to occur in genome-wide methylation patterns, but not in chromatin accessibility status in early preimplantation embryos (Guo et al., 2017; Wang et al., 2021), while Argelaguet *et al.* found regulatory elements associated with each germ layer to be epigenetically primed or remodeled before cell-fate decisions in gastrulating embryos (Argelaguet et al., 2019). In line with these results, Clark *et al.* found coupling between epigenetic layers to increase as cells commit to downstream lineages across embryonic stem cell differentiation (Clark et al., 2018). Using sc-multiomics approaches, several groups have characterized the dynamics between chromatin accessibility and gene expression in the same cell, in diverse lineage commitment contexts: from hair follicle differentiation (Chen et al., 2019) to neuronal differentiation of intermediate progenitors in the cortex (Ma Sai et al., 2020) and oligodendrocyte maturation (Xu et al., 2022). Leveraging the power of such approaches, chromatin accessibility was found to precede gene expression increases at lineage-specifying genes during lineage-commitment (Ma Anjun et al., 2020; Xu et al., 2022). Ma *et al.* showed this primed chromatin state can be used as a quantitative measure and a predictor of cell fate. Thus, sc-multiomics approaches find numerous applications in biological studies, providing unprecedented insight into mechanisms of gene regulation underlying cell identity and fate.

In this review, we focus, in particular, on the most recent techniques that integrate genome, epigenome and transcriptome profiling. All these techniques are discussed in detail in the sections below and summarized in Table 1. One molecular layer that is also tackled by single-cell multiomics techniques, but which we do not cover in detail here is the cell proteome. The most recently developed sc-multiomics methods that profile cell proteins in addition to at least one other molecular layer are summarized in Box 1.

**TABLE 1:** Summary of reviewed single-cell multiomics methods.

Method	Molecular layers profiled					Throughput (low/medium/high)	Special features (compared to techniques from same category)	Format	References
	Epigenome			Genome	Transcriptome				
	Chromatin accessibility	Chromatin conformation	DNAme	CNVs/ploidy/microsatellites/mutation	poly(A)+ RNA				
*scCAT-seq*	x				x	+	⇑ usable fragments	well	Liu et al. (2019)
*Paired-seq*	x				x	+++	⇑ throughput	well	Zhu et al. (2019)
*sc(ATAC + RNA)-seq*	x				x	+	⇓ cost; simple workflow	well	Reyes et al. (2019a)
*sci-CAR*	x				x	+++	⇑ acc. & RNA intersect coverage	well	Cao et al. (2018)
*SNARE-seq*	x				x	+++	⇑ sensitivity	droplet	Chen et al. (2019)
*ASTAR-seq*	x				x	++	⇓ price-performance ratio	microfluidics	Xing et al. (2020)
*SHARE-seq*	x				x	+++	⇑ throughput, performance	well	Ma Sai. et al. (2020)
*ISSAAC-seq*	x				x	+++	⇑ throughput, performance (esp. ATAC)	well/droplet	Xu et al. (2022)
*scDam&T-seq*		x			x	+	protein-DNA interactions information	well	Rooijers et al. (2019)
*scNOMe-seq*	x		x			+	estimates nucleosome phasing	well	Pott, (2017)
*scCOOL-seq*	x		x	x		+	⇑ acc. & DNAme intersect coverage	well	Guo et al. (2017)
*iscCOOL-seq*	x		x			++	⇑ accessibility coverage	well	Gu et al. (2019a)
*scMethyl-HiC*		x	x			+	⇑ mapping rate	well	Li et al. (2019)
*sn-m3C-seq*		x	x			+++	⇑ DNAme coverage	well	Lee et al. (2019)
*scNMT-seq*	x		x		x	++	⇑ throughput	well	Clark et al. (2018)
*scNOMeRe-seq*	x		x		x	+	⇑ DNAme coverage	well	Wang et al. (2021)
*scSIDR-seq*				x	x	+	captures total RNA	well	Han et al. (2018)
*TARGET-seq*				x	x	+++	⇓ cost; ⇑ throughput	well	Rodriguez-Meira et al. (2019)
*RETrace*			x	x		+	captures microsatellites	well	Wei and Zhang, (2020)
*scTrio-seq2*			x	x	x	++	⇑ DNAme coverage	well	Bian et al. (2018)

Throughput: + <500 cells, ++ <2000 cells, +++ >2000 cells. acc. = (chromatin) accessibility. scCAT-seq, single-cell chromatin accessibility and transcriptome sequencing), Paired-seq - parallel analysis of individual cells for RNA, expression and DNA, accessibility by sequencing, sc(ATAC + RNA)-seq - simultaneous chromatin accessibility and gene expression profiling, sci-CAR, single-cell combinatorial indexing-based chromatin accessibility and RNA, SNARE-seq, single-nucleus chromatin accessibility and mRNA, expression profiling; ASTAR-seq, assay for single-cell transcriptome and accessibility regions; SHARE-seq, simultaneous high-throughput ATAC, and RNA, expression with sequencing in single cells; ISSAAC-seq, *in situ* SHERRY, after ATAC-seq, scDam&T-seq - single-cell DNA, adenine methyltransferase identification (DamID) and messenger RNA, sequencing, scNOMe-seq - single-cell nucleosome occupancy and methylome-sequencing, scCOOL-seq, single-cell chromatin overall omic-scale landscape sequencing, iscCOOL-seq, improved single-cell chromatin overall omic-scale landscape sequencing; scMethyl-HiC, single-cell DNA, methylation and chromatin conformation capture, sn-m3C-seq - single-nucleus methyl-chromatin conformation capture sequencing, scNMT-seq, single-cell nucleosome, methylation and transcription sequencing, scNOMeRe-seq - single-cell nucleosome occupancy, methylome and RNA, expression sequencing; scSIDR-seq, simultaneous isolation and sequencing of genomic DNA, and total RNA, TARGET-seq, single-cell targeted mutational analysis and parallel RNA, sequencing, RETrace - simultaneous retrospective lineage tracing and methylation profiling of single cells, scTrio-seq - single-cell triple omics sequencing.

**Box 1:** Recent developments in single-cell multiomics proteome profiling.

Method	Molecular layers profiled	Proteins profiled	Throughput	References
*inCITE-seq*	proteins and RNA	intranuclear	+++	Chung et al., 2021
*PHAGE-ATAC*	proteins and chromatin	surface and intracellular	+++	Fiskin et al., 2021
*TEA-seq*	proteins, RNA and chromatin	surface	+++	Swanson et al., 2021
*DOGMA-seq*	proteins, RNA and chromatin	surface and intracellular	+++	Mimitou et al., 2021

Throughput: +++ > 2,000 cells. chromatin = chromatin accessibility.

### 3.1 Chromatin and Transcriptome

RNA sequencing has been a widely used application of next-generation sequencing. While traditionally researchers have been focused on quantifying gene expression, transcriptomic studies also provide valuable insight into processes such as alternative splicing events, or gene regulation by non-coding RNAs, such as enhancer RNAs. The physical accessibility of chromatin to regulatory factors, such as RNA polymerase or transcription factors, determines the likelihood that each specific region in the genome will get transcribed and expressed. Thus, the ability to probe nucleosome packing is also crucial to understanding transcriptional regulation.

The combined analysis of chromatin accessibility and transcriptome in the same cell provides a direct link between the state of chromatin at specific genomic regions and the level of the corresponding transcripts. Furthermore, signals from different gene regulatory processes are characterized by different temporal dynamics. While transcription factor binding is transient, transcriptional responses unfold across minutes to hours, and changes in epigenetic marks reflected at the level of chromatin accessibility can last for days and weeks (Reyes et al., 2019; Ma Sai et al., 2020) Thus, techniques that allow for simultaneous interrogation of chromatin accessibility and gene expression facilitate the dissection of cell states across different timescales (Reyes et al., 2019; Ma Anjun et al., 2020). To identify regions of open chromatin, a recent approach is to use the Tn5 transposase. This enzyme cleaves the DNA and inserts sequencing adapters at accessible genomic sites (in-between nucleosomes), allowing tagmentation and amplification of accessible chromatin. These regions can be easily identified after sequencing, using the inserted adapters.

The recently developed techniques for simultaneous profiling of chromatin and transcriptome we review are summarized in Table 1 and include:

(A) well-based low-throughput methods such as sc (ATAC+RNA)seq (Reyes et al., 2019b), scCAT-seq (Liu et al., 2019) and scDam&T-seq (Rooijers et al., 2019).
(B) ASTAR-seq (Xing et al., 2020), a continuous-flow microfluidics approach of higher throughput,
(C) Paired-seq (Zhu et al., 2019), sci-CAR (Cao et al., 2018), SNARE-seq (Chen et al., 2019), SHARE-seq (Ma Sai et al., 2020) and ISSAAC-seq (Xu et al., 2022), which are high-throughput methods that allow profiling of tens of thousands, up to millions of single cells, rendering them suitable for organismal-scale measurements.

#### 3.1.1 Low-Throughput Methods

Reyes *et al.* described a simple and cost-efficient method for assaying chromatin accessibility and the transcriptome of single cells, referred to in our text as sc (ATAC + RNA)-seq (Reyes et al., 2019b). In this approach, fixed cells are permeabilized and tagmented in bulk to minimize time and costs associated with this step. FACS-isolated single cells are then lysed, poly(A)+ RNAs are isolated using oligo-dT beads and reverse transcribed. The resulting cDNA is amplified and RNA-seq libraries are prepared using the Smart-seq2 protocol. Separately, the tagmented gDNA is amplified using indexed PCR to prepare ATAC-seq libraries. According to the authors, the sc (ATAC + RNA)-seq chromatin accessibility data showed similar quality and the transcriptomic data showed similar gene counts as comparable datasets generated using unimodal single-cell techniques. However, the RNA-seq data was found to be enriched for intronic reads, suggesting some capture of nuclear, nascent poly(A)+ RNAs. This can be disadvantageous if the aim of the experimenter is capturing only poly(A)+ RNA because the contaminant immature RNAs will lead to a decreased amount of usable reads for the poly(A)+ RNAs. Using sc (ATAC + RNA)-seq Reyes *et al.* successfully showed clustering of three different immune cell types and linked chromatin accessibility at regulatory elements to expression of their target genes. However, the clustering performance of this technique is lower when compared to mono-omics scRNA-seq approaches, with the resulting data quality limiting its applicability towards unbiased discovery of marker genes, despite the high number of genes detected (1,500–3,000 genes per cell). This could likely be improved by either adopting an alternative clustering approach, or by optimizing the separation of DNA from RNA, and by DNase treatment of the RNA, which could decrease the contamination of gDNA and nuclear RNAs (Reyes et al., 2019a).

scCAT-seq (Liu et al., 2019) is an efficient, low-throughput technique which involves partial lysis of FACS-isolated single cells and separation of the cytosolic RNAs from the DNA using centrifugation. Smart-seq2 is thereafter used for RNA-seq library preparation, and Tn5 is used to tagment the gDNA and profile chromatin accessibility. Using scCAT-seq, the authors successfully generated the first single-cell integrated map of chromatin accessibility and transcriptome in human embryos across preimplantation development. Using co-variability between accessibility of regulatory elements and significant gene expression changes, Liu *et al.* defined cell-specific regulatory relationships and identified cell-specific master transcription factors (TF). scCAT-seq accessibility data showed a reduction of mitochondrial reads compared to bulk ATAC-seq. In comparison to scATAC-seq, scCAT-seq provided more usable fragments–fragments which map uniquely to the genome and are not discarded by duplicate removal. However, in scCAT-seq, a smaller fraction of the usable fragments fell into peak regions, leading to a lower signal-to-noise ratio compared to scATAC-seq. The RNA-seq coverage was comparable to Smart-seq scRNA-seq. Finally, scCAT-seq is more expensive than sc (ATAC + RNA)-seq, due to the large number of individual transposition reactions.

scDam&T-seq (Rooijers et al., 2019) was the first method to simultaneously profile the transcriptome and protein-DNA interactions in single cells. scDam&T-seq uses the DamID approach in which cells are first transfected with a fusion between a Dam methyltransferase and a target protein, leading to accumulation of m6A marks at accessible sites in the gDNA. As such, protein-DNA interactions (usually indicative of accessible chromatin regions) are measured across time. Under scDam&T-seq, after lysis and reverse transcription (RT), individual cells are isolated by FACS and subjected to DpnI restriction, digestion, leading to fragmentation of the gDNA at m6A sites only and resulting in fragments that were inaccessible to methylation. Subsequently, cDNA and gDNA are barcoded and amplified during library preparation. Rooijers *et al.* applied scDam&T-seq to probe the nuclear organization of chromatin in mouse embryonic stem cells (mESCs). The authors showed that genomic regions within the nucleoplasm are more likely to be actively transcribed in cells in which these chromatin sites are detached from the nuclear lamina. scDam&T-seq minimizes loss of material and technical bias by omitting nucleotide separation and linear amplification. Compared to the triple-omics scNMT-seq (Clark et al., 2018) (discussed in the following sections), scDam&T-seq shows similar RNA-seq and chromatin accessibility data quality, but requires much lower sequencing depth which also reduces the overall costs. One limitation of the protocol is the need for transfection of the cells with the Dam constructs, which may additionally present with variabilities in efficiency depending on the cell types of interest.

#### 3.1.2 High-Throughput Methods

ASTAR-seq (Xing et al., 2020) uses the Fluidigm C1 microfluidic platform to isolate and lyse cells, reverse transcribe, tagment, and finally amplify the cDNA and gDNA. During amplification, the cDNA is biotinylated, which allows for its subsequent separation from gDNA using streptavidin beads. Libraries are prepared separately for the gDNA and cDNA off the chip. Using ASTAR-seq, Xing *et al.* arranged primary cells along the erythroblast differentiation pseudotemporal trajectory and identified genes and regulatory elements responsible for the progression of differentiation. Compared to scCAT-seq, ASTAR-seq captures the whole-cell transcriptome, shows higher genomic alignment rates, and higher gene detection rates at a comparable library complexity. Furthermore, ASTAR-seq had the highest sensitivity in transcriptomic data among all similar techniques with a very high rate of libraries detecting more than 15% of genes and mapping more than 75% to exons. In addition, the chromatin accessibility data showed significantly higher signal-to-noise ratio than unimodal scATAC-seq libraries. However, ASTAR-seq requires the Fluidigm microfluidic platform, which can limit the customization to specific needs and increase costs depending on the number of cells profiled.

Among the high-throughput methods, sci-CAR (Cao et al., 2018) uses a well-based combinatorial indexing strategy with two rounds of barcoding. The first barcoding occurs during reverse transcription and tagmentation, after which the nuclei are sorted by FACS and the lysate is split in two and indexed with different primers for separate library preparation of cDNA and gDNA. Cao *et al.* identified distinct cell types present in a whole-kidney primary cell culture system and linked the chromatin accessibility of distal *cis*-regulatory elements (CREs) to their target gene expression changes by using sci-CAR. This approach requires custom synthesis of an array of indexed reagents, which increases throughput but can prove costly and time intensive. In addition, as with other high-throughput techniques, library complexity was not as high as for low-throughput methodologies, especially with regards to chromatin accessibility data.

Paired-seq (Zhu et al., 2019) is an ultra-high-throughput well-based method that makes use of a limiting dilution approach to obtain single cells in individual wells, and applies a ligation-based combinatorial sequencing strategy using five barcoding rounds. The first barcoding occurs during the *in situ* RT and tagmentation, followed by three rounds of split-and-pool barcoding ligations. A final round of barcoding during DNA tailing is followed by additional amplification. This multi-step barcoding approach drastically increases the throughput of the technique. Amplicons are then split, and each aliquot is digested by a different enzyme leading to dedicated libraries for genome and transcriptome sequencing. Zhu *et al.* showed that Paired-seq could identify major cell types in the adult mouse cerebral cortex, reconstruct the trajectory of cellular lineages within the developing forebrain, and link distal CREs to potential target genes. While allowing for very high throughput, the lengthy barcoding process of Paired-seq leads to a low nuclear recovery rate, potentially precluding its applicability to low-input or rare samples, such as clinical specimens. Paired-seq showed lower coverage than the corresponding mono-omics techniques for both the transcriptome and chromatin accessibility data.

SNARE-seq (Chen et al., 2019) is a droplet-based method where cell nuclei are permeabilized and tagmented in bulk, then individually encapsulated in microdroplets, using a microfluidic device, for simultaneous barcoding of gDNA fragments and mRNA from the same cell. The encapsulated nuclei are then lysed, reverse transcribed, and amplified, after which the amplicons are split into aliquots for separate library preparation. Using SNARE-seq, Chen *et al.* identified major and rare cell types in the mouse neonatal cerebral cortex. They managed to capture finer distinctions between closely related cellular states than by previously generated scRNA-seq, and revealed linked gene expression and chromatin accessibility dynamics during neurogenesis. SNARE-seq shows increased sensitivity for accessible sites compared to sci-CAR, and RNA-seq sensitivity similar to that of single-nucleus droplet-based RNA-seq (snDrop-Seq (Lake et al., 2019)) (see Table 2). Comparable to sci-CAR and Paired-seq, SNARE-seq only profiles the nuclear transcriptome, leading to an enrichment for intronic regions in the transcriptomic data.

**TABLE 2:** Performance in data quality and coverage achieved by the single-cell multiomics techniques reviewed.

Method	Genome data		Transcriptome data			DNA methylome data	
	Coverage (# of accessible regions captured per cell)	Mapping rate	Gene detection rate	Exon mapping rate	Mapping rate	Coverage (# of CpGs captured per cell)	Mapping rate
*scCAT-seq*	210,000 uniquely mapped fragments/cell	67%	8,725 (human)		54.9%		
*Paired-seq*	2,114 unique reads/nucleus; 1,367 ATAC fragments in peaks	-	1,481 unique reads/nucleus; 726 UMIs (mouse)	-	-		
*sc(ATAC + RNA)-seq*	1,000–10,000 unique fragments/cell; 40–65% map to peaks	-	1,500–3,000 genes/cell (human)	1.6%	61%		
*sci-CAR*	1,456 unique reads; 915 ATAC fragments in peaks	-	3,276 UMIs (mouse)	-	-		
*SNARE-seq*	2,720/nucleus; 1,059 ATAC fragments in peaks	91%	623 UMIs (mouse)	37%	94%		
*ASTAR-seq*	142,886 library size; 27.9% fragments in peaks	86%	>15% (9,739) (human)	>75%	73.8%		
*SHARE-seq*	7,805 ATAC fragments in peaks; 65.5% fragments in peaks	-	9,290 UMIs (mouse)	-	-		
*ISSAAC-seq*	58,000 unique reads in peaks; 37% fragments in peaks		>17,000 UMIs (mouse); >4,000 genes (mouse)	35–60%			
*scDam&T-seq*	-	-	2,282 genes (mouse)	-	-		
*scNOMe-seq*	6.7 million GpCs/cell; 20,388 DHSs	52%				1.3 million CpGs/cell	-
*scCOOL-seq*	2,800 NDRs/cell; 19.7 million GCH; aggregate: 77.2%	22%				2.2 million WCGs (10.1%); agg.: 67.4%	-
*iscCOOL-seq*	Aggregate: 84.7% of GCH	62%				Aggregate: >80% of CpG sites	-
*scMethyl-HiC*	80,763 informative contacts per nucleus (150 cells)	-				567,380 CpGs/nucleus	-
*sn-m3C-seq*	500,000 contacts/cell (4,200 cells)	-				27.5% of mouse genome	72%
*scNMT-seq*	15% of GpCs; 75% of promoters, 85% of gene bodies probed	-	-	-	-	22.8% of mouse genome	32%
*scNOMeRe-seq*	31 million GCH; 15.5% per cell	-	10,000–15,000 genes (mouse)	-	-	3.49 million WCG (15.8%)	-
*scSIDR-seq*	-	>90%	5,690 genes (human)	87%	-		
*TARGET-seq*	Detected all mutations in 98.4% of cells	-	8,200 genes (human)	-	-		
*RETrace*	1,217 microsatellites/cell	-				146,000 CpGs/cell	-
*scTrio-seq*	-		8,106 genes (human)	-	-	16.4 million CpGs/cell	-

Grey blocks = molecular layer not profiled by the technique. - = molecular layer is profiled by the technique, but data is not provided. The methylation status of GCH, sites (GCA/GCT/GCC) is used to analyze chromatin accessibility, while the methylation status of WCG, sites (ACG/TCG) is used to analyze the endogenous DNA, methylation.

SHARE-seq (Ma Anjun et al., 2020) is a high-throughput method, which builds on Paired-seq, associated with low costs and superior transcriptomic profiling performance. As with Paired-seq, SHARE-seq relies on repeated rounds of hybridization-based barcoding. In SHARE-seq, unlike for Paired-seq, cells are fixed before being permeabilized, so SHARE-seq relies on the split-pool ligation-based transcriptome (SPLiT-seq) (Rosenberg et al., 2018) library preparation strategy, which is compatible with fixed cells. Following permeabilization, SHARE-seq subjects cells to gDNA tagmentation and RT using biotin-tagged poly-dT primers, which are performed in bulk. Single cells are then isolated by limiting dilution, then subjected to three rounds of hybridization barcoding. Finally, cDNA is separated from gDNA fragments using streptavidin beads and each library is prepared for sequencing. Using peak-gene associations revealed by SHARE-seq, Ma *et al.* identified genomic regions of regulatory chromatin, whose accessibility is established prior to the target gene’s expression, reflecting a mechanism of lineage priming in the context of hair follicle differentiation. SHARE-seq showed a higher fraction of fragments occurring in peaks and a higher RNA library complexity than sci-CAR, SNARE-seq and Paired-seq (see Table 2).

ISSAAC-seq (Xu et al., 2022) is the most recent technique for simultaneous profiling of chromatin accessibility and transcriptome in single cells that we review here. ISSAAC-seq is the first sc-multiomics technique to apply the Sequencing HEteRo RNA-DNA-hYbrid (SHERRY) (Di et al., 2020) approach for transcriptomic library preparation. Thus, for ISSAAC-seq, nuclei are first subjected in bulk to chromatin tagmentation by Tn5 loaded with Nextera adapters, then to RT with a primer containing a partial TruSeq adaptor, a UMI and a poly-T sequence. Then, a Tn5 loaded with one side of the Nextera sequence is added to the ISSAAC-seq reaction to specifically tagment the DNA-RNA heteroduplexes resulted from RT, according to the SHERRY approach, followed by exonuclease I digestion and gap fill-in reaction. Next, nuclei can be isolated either by FACS or by droplet microfluidics, barcoded and pooled again. Fragments are pre-amplified and the library is split into two aliquots, and amplified independently for the two library types. Xu *et al.* applied ISSAAC-seq in the mouse cerebral cortex to investigate the dynamics of chromatin accessibility and gene expression during oligodendrocyte maturation. ISSAAC-seq has a flexible workflow, which facilitates its transferability to both high and low throughput applications. It also shows better performance and sensitivity than similar approaches, such as sciCAR-seq, SNARE-seq, Paired-seq and SHARE-seq, with an exceptionally high fraction of ATAC fragments occurring in peaks.

Linking open chromatin dynamics with gene expression changes has proven one of the most popular goals in multiomics. The quick implementation of these methods by other research groups was facilitated by the fact that the processing for bulk ATAC-seq can be readily adapted for single-cells by experienced users. However, given the numerous recent advances in single-cell parallel chromatin and transcriptome profiling reviewed in this section (see Table 1), experimenters are faced with an especially challenging task when searching for the optimal technique of choice. Among the low-throughput approaches, sc (ATAC + RNA)-seq is associated with the lowest costs and simplest workflow. In contrast, ASTAR-seq offers superior gene detection and alignment rates, and a good price-performance ratio, but requires access to a microfluidic platform. Among the high-throughput approaches, Paired-seq has the highest throughput, while SHARE-seq and ISSAAC-seq achieve superior performance. As is the case for profiling of other molecular layers, the lower-throughput approaches such as ASTAR-seq and scCAT-seq detected roughly 10 times more genes and 100 times more accessible sites than sci-CAR, SNARE-seq and Paired-seq (see Table 2). Exceptions are SHARE-seq and ISSAAC-seq, which enable high throughput and very high performance in the number of genes/accessible chromatin sites identified, approaching the analytical power of low-throughput techniques. All techniques reviewed in this section are limited to profiling poly(A)+ RNAs, with all five high-throughput techniques being restricted to only nuclear poly(A)+ RNA. Among the others, scCAT-seq profiles only cytoplasmic poly(A)+ RNAs, while ASTAR-seq profiles the whole-cell poly(A)+ RNAs.

### 3.2 Chromatin and DNA Methylation

Sites in the genome that are accessible to DNA-cleaving enzymes such as Tn5 or DNase I are considered to correspond to areas of decondensed chromatin, which are functionally associated with increased transcriptional activity and are referred to as DNase I hypersensitive sites (DHS). The ability to simultaneously assess chromatin structure and DNA methylation (DNAme) status in single cells provides insight into the functional interaction between these two distinct regulatory layers and their contribution to shaping gene expression. Techniques that have recently been developed focus on combining DNAme characterization either with chromatin accessibility profiling such as scCOOL-seq (Guo et al., 2017), its higher-throughput derivative—iscCOOL-seq (Gu et al., 2019b) and scNOMe-seq (Pott, 2017), or with profiling the large-scale 3D chromatin conformation as is the case for scMethyl-HiC (Li et al., 2019) and sn-m3C-seq (Lee et al., 2019). These techniques are summarized in Table 1.

Common to scCOOL-seq, iscCOOL-seq, scNOMe-seq (discussed in this section), scNOMeRe-seq (Wang et al., 2021) and scNMT-seq (Clark et al., 2018) (discussed in the following section) is the NOMe-seq strategy, where the M. CviPI enzyme is utilized to methylate accessible GpC sites in the genome, creating a footprint of nucleosome-free chromatin regions. All these methods show high resolution of open chromatin profiling within individual loci, and footprinting of transcription factors. In addition, they control for fragment loss, making it possible to distinguish between the undetected and the closed chromatin states (especially important in single cells due to allelic dropout) (Pott, 2017). However, this means there is no *a priori* enrichment for accessible chromatin, in contrast to count-based methods, such as ATAC-seq, and, therefore, significantly deeper sequencing is required to achieve similar coverage (Pott, 2017). In addition, not all DHSs captured by ATAC-seq and DNase-seq are detectable using the NOMe-seq strategy-based techniques because chromatin accessibility measurement relies on GpC occurrence within DHSs (Pott, 2017).

scCOOL-seq (Guo et al., 2017) was the first technique to profile chromatin accessibility and DNA methylation in single cells. In addition, scCOOL-seq is also able to detect copy number variations (CNVs) and cell ploidy. First, single cells are manually picked, lysed and treated with M. CviPI, followed by protease digestion to remove DNA-bound proteins. Next, gDNA is bisulfite-converted and libraries are prepared using the post-bisulfite adapter tagging (PBAT) protocol (Miura et al., 2012), which includes adapter tagging and two rounds of random primer extension. Guo *et al.* applied scCOOL-seq to study allele-specific epigenetic reprogramming during mouse preimplantation embryonic development. They found that, up to the blastocyst stage, embryos showed differences in DNA methylation patterns between alleles but not in accessibility and that, at the two-cell stage, there was evidence of epigenetic priming of pluripotency factors. Of note, scCOOL-seq showed promising intersect coverage, identifying chromatin accessibility and DNA methylation status for more than 70% of the investigated promoters.

scNOMe-seq is an adaptation of the bulk NOMe-seq assay (Kelly et al., 2012) for chromatin accessibility and DNA methylation (Pott, 2017). In this technique, cells are lysed and homogenized to release their nuclei, which are then incubated with GpC methyltransferase in bulk. Then, individual nuclei are isolated by FACS and lysed to release gDNA, which is bisulfite converted and used for PBAT library preparation. scNOMe-seq was used to measure chromatin accessibility at DHSs and TF binding sites, and assess nucleosome phasing in GM12878 and K562 cells. Although scNOME-seq clustered cells according to cell type, a very low amount of all GpCs (2.9%) and CpGs (3.6%) in the genome were covered in each cell, in comparison to single-cell whole-genome bisulfite sequencing (scWGBS-seq (Smallwood et al., 2014)), which provides up to 20% coverage in each individual cell (Smallwood et al., 2014).

iscCOOL-seq (Gu et al., 2019b) is an improved version of scCOOL-seq, developed by the same research group. scCOOL-seq, scNOMe-seq, and scNMT-seq all rely on the PBAT library preparation strategy (Guo et al., 2017) that includes two random priming steps leading to a high amount of unmappable reads. In contrast, iscCOOL-seq uses a simplified Tailing- and Ligation-free (TAILS) library construction strategy including only one random priming step, which offers an increased mapping efficiency. Gu *et al.* used iscCOOL-seq in combination with scRNA-seq to characterize mouse oocyte maturation and found that the most dramatic changes in chromatin accessibility occur during growth initiation, along with gene expression alterations, and increased variability in DNA methylation levels between individual oocytes. In addition, highly expressed genes across oocyte growth showed nucleosome-depleted regions (NDRs) at their transcription end sites (TESs). iscCOOL-seq shows improved coverage compared to the other techniques described so far in this section (see Table 2).

All of the methods described above focus on the local chromatin and DNA properties of the genome. However, they do not detect interactions between distal genomic regions which can become proximally close through higher-order chromatin structures, such as chromatin loops and topologically associating domains (TADs) (Rowley and Corces, 2018). These higher-order features recently emerged as important gene regulation mechanisms, which allow for coordinated DNA methylation to occur across large genomic distances (Rowley and Corces, 2018). Thus, methods for simultaneous profiling of chromatin 3D conformation and DNA methylation have the potential to reveal novel layers of gene expression regulation. Chromatin 3D conformation detection techniques such as Hi-C have been increasingly used in bulk cell systems to reveal long-range regulatory interactions. Hi-C allows sequencing of fragments that are brought in close physical proximity through chromatin looping by crosslinking DNA-protein complexes, followed by fragmentation, extraction, ligation, and digestion of the genomic DNA with restriction enzymes (Li et al., 2019). Applied to sc-multiomics, two methods recently emerged which combine Hi-C and bisulfite conversion, providing concomitant information about contacts between distant genomic loci and their DNA methylation status in individual cells.

In scMethyl-HiC (Li et al., 2019), cells are crosslinked and lysed, nuclei are permeabilized and subjected to restriction digestion by DpnII. The resulting DNA fragments are biotinylated and proximally ligated, with all steps conducted in bulk. Subsequently, single nuclei are isolated by FACS and subjected to bisulfite conversion, random priming, adapter ligation, and library amplification. Using scMethyl-HiC, Li *et al.* clustered mESCs cultured in different media and characterized cluster-specific chromosome conformation and coordinated DNA methylation at chromatin loop anchors. The authors captured similar numbers of CpGs and of informative contacts per nucleus using scMethyl-HiC as with mono-omics single-cell methods, but scMethyl-HiC was limited by its low throughput.

sn-m3C-seq (Lee et al., 2019) shares a similar workflow to scMethyl-HiC, but instead of biotinylation of the digested DNA fragments, it applies an improved single-nucleus methylcytosine sequencing (snmC-seq2) library preparation strategy (Luo et al., 2018). This maximizes mapping rates, increases throughput, and improves library complexity by optimizing the sequence of the primers and the nucleotide concentration used in the random-primed DNA synthesis reactions. Lee *et al.* used sn-m3C-seq to identify distinct cell-type specific 3D chromatin structures in the human frontal cortex, which were associated with differential DNA methylation signatures. In addition, they found that genes were more likely to be active when a TAD boundary was present at their promoters. Compared to scNMT-seq (discussed below), sc-m3C-seq showed a two-fold higher read mapping rate, similar library complexity, but lower bias for CpG islands, while the chromatin conformation data quality was comparable to that of single-cell unimodal datasets.

DNA methylation remains the most explored epigenetic mark and single-cell techniques have adopted the commonly used bisulfite treatment to identify methylated CpGs. However, bisulfite conversion causes wide degradation of DNA fragments, and other types of base modifications such as hydroxymethylation remain elusive. The recent technological advances reviewed in this section provide researchers the possibility to combine whole-genome profiling of DNA methylation in single cells with interrogation of chromatin states. Among the methods discussed in this section, iscCOOL-seq (for chromatin accessibility) and snm3C-seq (for chromatin conformation) have the highest throughput. Regarding chromatin accessibility data, iscCOOL-seq offers the highest coverage of GpC sites in the genome at decreased sequencing costs. sn-m3C-seq showed the deepest coverage of DNA methylation data, while sc-Methyl-HiC showed the highest genome mapping rates. Regarding chromatin conformation data, sn-m3C-seq detected more informative contacts per cell than the other reviewed methods.

### 3.3 Chromatin, DNA Methylation, and Transcriptome

Open chromatin provides a dynamic platform through which epigenetic factors can act in a cooperative fashion to regulate gene expression in cells. Thus, the ability to understand how these regulatory domains are established in single cells and contribute to gene expression regulation would achieve a uniquely comprehensive picture of the regulatory mechanisms of gene expression. scNMT-seq (Clark et al., 2018) and scNOMeRE-seq (Wang et al., 2021) are two powerful triple-omics approaches that profile chromatin accessibility, DNA methylation, and the transcriptome simultaneously in single cells (summarized in Table 1).

scNMT-seq (Clark et al., 2018) was the first method to characterize the transcriptome together with chromatin accessibility and DNA methylation in single cells. Under scNMT-seq, following FACS, cell lysis and GpC methyltransferase treatment, RNAs are separated using oligo-dT beads and used for Smart-seq2, while the gDNA is bisulfite converted and used for scBS-seq library preparation. By applying scNMT-seq, Clark et al. constructed the first triple-omics atlas of mouse gastrulation in which novel temporal lineage-specific epigenetic patterns were revealed. Furthermore, the authors found that the initial exit from pluripotency was associated with establishment of a global repressive epigenetic landscape and lineage-specific epigenetic patterns.

Wang *et al.* used scNOMeRe-seq (Wang et al., 2021) to build the first single-cell triple-omics map of mouse preimplantation development. The method uses manual cell picking to isolate single cells, and combines two previously established techniques: scNOMe-seq, for characterization of the chromatin and DNAme landscapes, and MATQ-seq, for transcriptome profiling. Unlike SmartSeq2 and other SMART-chemistry based methods, MATQ-seq uses optimized primers which improve the efficiency of both first- and second-strand synthesis. In addition, unique molecular identifiers (UMIs) are used to pre-label each unique molecule and help to address a potential bias introduced during amplification. Notably, scNOMeRe-seq showed increased sensitivity for detection of lowly expressed genes and even coverage through genic regions.

The ability to simultaneously profile three molecular layers at single-cell resolution greatly improves the study of highly complex molecular events and delivers novel insight into the role of the epigenome in lineage commitment and epigenetic reprogramming events during embryonic development. Crucially, data quality is not compromised by expanding single-cell techniques to conduct triple-omics studies. Thus, scNMT-seq and scNOMeRe-seq both show comparable or even superior coverage of chromatin accessibility and methylome data compared to the dual-omics techniques from the previous section, but lower alignment rates (see Table 2). scNOMeRe-seq also showed increased sensitivity for lowly expressed genes when compared to mono-omics scRNA-seq datasets. While scNMT-seq provides higher throughput, scNOMeRe-seq excelled in higher methylome coverage. While providing additional information, these techniques are naturally associated with a lengthier workflow and higher sequencing costs when compared to the other reviewed methods.

## 4 Integrated Profiling of Genetic Variation and Gene Expression or DNA Methylation in Single Cells

Genomic heterogeneity is generally associated with cancerous cells but genetic mosaicism also occurs under physiological conditions in healthy tissues, including immune cells (Han et al., 2018) and neurons (Rohrback et al., 2018), with mostly unexplored functional consequences. In this section, we discuss methods that characterize genetic variation and their potential effects on the transcriptome or DNA methylome of individual cells (summarized in Table 1).

scSIDR-seq (Han et al., 2018) is a low-throughput approach for whole-genome and total transcriptome profiling, which accurately captures CNVs and single-nucleotide polymorphisms (SNPs). In scSIDR-seq, cells are initially bound to anti-EpCAM antibody-conjugated magnetic beads and individually isolated in wells by limiting dilution. Thereafter, cells are subjected to hypotonic lysis, which preserves the integrity of the nuclear lamina and releases total RNA (cytoplasmic and nuclear) into solution. Using a magnet, the bead-bound gDNA is retained in the pellet and processed separately from the supernatant containing total RNA. scSIDR-seq was employed by the authors to distinguish cells from different tumor cell lines using their CNV and gene expression profiles. Furthermore, the authors found strong global correlations between CNVs of genomic regions and the expression of nearby genes. Altogether, scSIDR-seq efficiently recovers DNA and RNA, shows high alignment rates (>90%), and can be adapted for the study of non-poly(A)+ long RNAs. However, the reliance of scSIDR-seq on cell-specific surface antibodies for capturing the cell lysate may require optimization if analysis of other cell types is desired.

TARGET-seq (Rodriguez-Meira et al., 2019) is a high-throughput technique for targeted, biallelic, mutational analysis and gene expression quantification in single cells. In TARGET-seq, cells are isolated by FACS and the cell lysis step includes a mild protease treatment, followed by protease inactivation, designed to improve the release of gDNA. Following RT, gDNA and cDNA are simultaneously pre-amplified using target-specific primers for known mutational hotspots, after which the amplicons are split for parallel library preparation. Rodriguez-Meira *et al.* developed an optimized cDNA library preparation protocol (Smart-seq+ (Rodriguez-Meira et al., 2019)) used for TARGET-seq, which minimizes allelic dropout rates and maximizes gene detection. The authors used TARGET-seq to analyze hematopoietic stem and progenitor cells (HSPCs) from the bone marrow of neoplasm patients and healthy controls. Using TARGET-seq, they resolved complex genetic subclones and found aberrant gene expression in non-mutant HSPCs, revealing likely tumor microenvironment effects on wild-type cells. TARGET-seq exhibits high sensitivity for detection of multiple mutations in the same cell, low library bias, is automated to a high degree, and does not require whole-genome amplification, resulting in reduced sequencing costs. However, it is only suitable for targeted mutational analysis, therefore it cannot be applied for the discovery of novel mutations, and its high throughput could only be reached for 3′-biased poly (A+) transcriptome libraries.

RETrace (Wei and Zhang, 2020) is a low-throughput approach that simultaneously captures microsatellite profiles and DNA methylation patterns, allowing single-cell retrospective lineage tracing, phylogenetic fate mapping, and cell type identification. To conduct RETrace, following isolation by FACS and lysis of cells, the DNA is fragmented by MspI and MseI restriction digestion. Next, the DNA is A-tailed, circularized, and split into two aliquots for methylation and microsatellite analysis: Mspl-resulting fragments are amplified by methylation PCR, while Msel-resulting fragments are PCR amplified and enriched for target microsatellite regions using hybridization probes. By applying RETrace, Wei and Zhang constructed an *ex vivo* retrospective lineage tree for HCT116 colorectal carcinoma cells with improved accuracy and with fewer cell divisions being required for detection of changes between cells. However, RETrace uses single-cell reduced-representation bisulfite sequencing (scRRBS (Guo et al., 2015)) and hybridization probe microsatellite capture (Gonzalez and Zardoya, 2013), resulting in relatively low genome coverage and precluding discovery of novel microsatellite loci. In addition, while RETrace enables retrospective lineage mapping in tissues exhibiting microsatellite unstable cells (e g., tumors), it would require significant adjustment for wider applicability to cells in healthy tissue, which exhibit much lower mutation rates.

scTrio-seq2 (Bian et al., 2018) was developed by building on the previously published scTrio-seq (Hou et al., 2016). scTrio-seq2 improved scTrio-seq by replacing scRRBS with scWGBS (Smallwood et al., 2014) and improving detection efficiencies. scTrio-seq2 relies on manual cell picking followed by magnetic bead-based separation of the nuclei from the cytosolic RNA and independent scRNA-seq and scWGBS library preparation. Bian *et al.* used scTrio-seq2 to reconstruct genetic lineages and concomitantly trace genomic and epigenomic effects on gene expression levels. They additionally revealed that, in colorectal cancer tumors, hypomethylated chromosomes also exhibit CNVs, and that transposable elements have aberrant DNA methylation compared to physiological development. scTrio-seq2 achieved comparable performance to mono-omics scRNA-seq and scBS-seq.

In this section, we reviewed sc-multiomics techniques that, instead of profiling chromatin status, analyze how genome variation, gene expression, and DNAme regulate each other. These genetic mutations are then correlated with transcriptomic data (scSIDR-seq, TARGET-seq), DNA methylome data (RETrace), or both (scTrio-seq2) to infer relationships between genetic and epigenomic or transcriptomic heterogeneity. This knowledge can then be applied toward the reconstruction of lineage trees, or the study of tumor microenvironment cell heterogeneity. Among the techniques discussed in this section, TARGET-seq offered the highest throughput, scSIDR-seq showed the highest alignment rates, while TARGET-seq and scTrio-seq2 showed the highest detection rates in their gene expression data. scTrio-seq2 also showed by far the highest DNA methylome data coverage among all the techniques reviewed (see Table 1 and Table 2).

## 5 Single-Cell Multiomics Data Analysis

While multiomics techniques with single-cell resolution can provide unique insight into cell-specific molecular processes, such data requires complex integration, raising important challenges for its computational analysis. The recent expansion of sc-multiomics techniques gave rise to the development of numerous pipelines designed for analysis of resulting datasets, which rely on different approaches.

Pipelines such as Seurat3 (Stuart et al., 2019) use correlation analysis between single-cell mono-omics data across cells. A second computational approach for sc-multiomics data analysis relies on one type of data to cluster subpopulations and integrates the other types of data onto the pre-specified clusters (Lee et al., 2020). For example, the sci-CAR developers used the RNA-seq data to cluster cells into subpopulations and then identified sites of open chromatin that were unique to each cluster (Cao et al., 2018). Lastly, Linked Inference of Genomic Experimental Relationships (LIGER (Welch et al., 2019)) and Multiomics Factor Analysis (MOFA (Argelaguet et al., 2018)) rely on the integrative analysis of all the modalities to generate the overall single-cell map, using matrix factorization approaches (Lee et al., 2020).

Several issues that concern sc-multiomics data analysis remain: the generation of massive amounts of data, which require intensive computational power and lengthy processing times; limited and non-standardized analytical functionalities of integrative tools, which complicate the interpretation and reproduction of results; and the current lack of robust benchmarking pipelines and integrative computational methods (Ma sai et al., 2020). Machine learning computational frameworks have already been applied towards classifying cells based on multiomics data integration (Autoencoder (Zhang et al., 2018), Amaretto (Champion et al., 2018)), identifying signature genes across developmental stages (EmPredictor (Liang et al., 2020)) and improving imputation (DeepImpute (Arisdakessian et al., 2019)) in single-cell RNA-seq data. Advancement in deep neural-network algorithms may also benefit sc-multiomics in the future by improving cell-subtype clustering, dropout imputation, and multiomics integration (Azad and Vafaee, 2019). Despite the multitude of novel tools available, biological discovery through integration of single-cell multi-layer data remains computationally challenging.

## 6 Discussion

sc-multiomics provides a multi-molecular readout that has already proven its potential for powerful and comprehensive dissection of the complex molecular mechanisms regulating gene expression for a more accurate depiction of individual cell states. sc-multiomics is particularly well-suited for applications that involve rare cell types, as it maximizes the information that can be obtained from each individual cell. Such approaches benefit from immense potential applications in a wide range of research fields, from developmental biology to cancer biology and precision medicine. sc-multiomics is, however, still a very young field, and the techniques that we have reviewed here are at the forefront of the most recent technical developments. Thus, many have so far been restricted to proof-of-concept applications, in contexts where the magnitude of the expected effects is considerably higher than for other biological systems in which more subtle differences are to be expected.

Important challenges in sc-multiomics that remain to be addressed include data sparsity and noise, gene or allelic dropout, high sequencing-associated costs, and low recovery efficiency from individual cells. The high costs associated with sequencing create a constant trade-off between throughput level and the richness of information afforded. This leads to a limited coverage per individual cell, creating data sparsity at several levels. For example, each molecular layer is profiled only at a fraction of the sites in the whole genome, and two molecular layers profiled together will not show concurrence at all sites within a cell; in addition, not all cells will have both layers profiled successfully. This presents an important challenge as the full potential of multiomics is only achieved when two or more epigenetic features of a genomic location can be intersected using data from the same cell. However, this will likely improve as sequencing costs will continue to decrease in the future.

Of note, most of the techniques for transcriptomic profiling reviewed here rely on oligo-dT capture of RNAs, which does not allow for capture of other non-poly (A+) RNAs with potential regulatory roles, such as short or long non-coding RNAs. However, future techniques may capitalize on recent developments such as Smart-seq-total (Isakova et al., 2020), where all RNA species within the cell are polyadenylated before oligo-dT capture. Such improvements may eventually allow the capture of total RNA to become the norm in sc-multiomics. In addition, the recently developed CELLO-seq (Berrens et al., 2020) allows long-read RNA-sequencing in single cells and may also soon be adopted by multiomics techniques. This would enrich the information we can extract from distinct levels of transcriptome organization by characterizing novel transcript isoforms at single-cell resolution. Another issue shared by most techniques is the abundance of sequenced reads from non-target regions, such as mitochondrial DNAs or ribosomal RNAs. The targeted depletion of undesired regions from the final libraries is a promising approach to improve the final sequencing results. Approaches such as loading Cas9 with guides against target regions have successfully been employed to deplete mitochondrial DNA fragments and ribosomal RNAs from libraries and may be a promising solution for sc-multiomics (Gu et al., 2016; Montefiori et al., 2017).

One aspect of sc-multiomics that is less developed is the profiling of DNA-associated proteins, including genome-wide mapping of histone modifications and transcription factor binding sites. Factors including lengthy processing and optimization associated with antibody-based techniques, as well as their dependence on high-quality commercially available antibodies, have mostly precluded the compatibility of techniques such as single-cell ChIP-seq (itChIP-seq) (Ai et al., 2019) with, for example, simultaneous transcriptome profiling. Two exceptions to this are scDam&T-seq (Rooijers et al., 2019) and single-cell calling cards (Moudgil et al., 2020), novel assays that allow simultaneous assessment of DNA-binding proteins and the transcriptome in single cells. However, both methods require genetic manipulation of the model cell or organism, precluding their application in clinical samples, for example. Only very recently have the first single-cell multiomics techniques for simultaneous profiling of histone modifications and transcriptome without genetic manipulation emerged. These techniques, Paired-Tag (Zhu et al., 2021) and CoTECH (Xiong et al., 2021), both apply combinatorial barcoding of cells to achieve high throughput and both rely on the use of the pA-Tn5 protein fusion for *in situ* antibody-targeted tethering of Tn5 to histone modification loci, similar to the mono-omic single cell technique CUT&Tag (Kaya-Okur et al., 2019). While so far these approaches have only been used to profile one individual histone mark at a time, the recent development of single-cell multiCUT&Tag (Gopalan et al., 2021) should allow the simultaneous profiling of multiple histone modifications and transcriptome in single cells in the near future. Despite such recent developments, so far, a single-cell multiomic technique that allows parallel interrogation of transcription factor binding sites and RNA is, to our knowledge, still lacking.

Finally, the power of sc-multiomics is expandable to other cell features, such as functional parameters, as in Patch-seq (Cadwell et al., 2016; Hu et al., 2018) which achieves single-neuron RNA-seq together with whole-cell electrophysiological patch-clamp recordings, and morphological characterization.

Still in its infancy, sc-multiomics presents many opportunities for optimization and advancement. Given the speed of technological innovation in biomedical research, sc-multiomics will surely continue to improve and become more widely adopted within the research community.
